# Radiosensitization of the PI3K inhibitor HS-173 through reduction of DNA damage repair in pancreatic cancer

**DOI:** 10.18632/oncotarget.22850

**Published:** 2017-12-01

**Authors:** Jung Hee Park, Kyung Hee Jung, Soo Jung Kim, Zhenghuan Fang, Hong Hua Yan, Mi Kwon Son, Juyoung Kim, Yeo Wool Kang, Ji Eun Lee, Boreum Han, Joo Han Lim, Soon-Sun Hong

**Affiliations:** ^1^ Department of Drug Development, College of Medicine, Inha University, Sinheung-dong, Jung-gu, Incheon 400-712, Republic of Korea; ^2^ Department of Internal Medicine, College of Medicine, Inha University, Sinheung-dong, Jung-gu, Incheon 400-712, Republic of Korea

**Keywords:** radiation, HS-173, DNA double-strand breaks (DSBs), DNA damage repair

## Abstract

Activation of PI3K/AKT pathway occurs frequently in tumors and is correlated with radioresistance. The PI3K/AKT pathway can be an important target for improvement of radiotherapy. Although adding of chemotherapy to radiation therapy regimen enhances survival in patients with locally advanced pancreatic cancer, more effective therapies for increasing radiosensitivity are urgently needed. In this study, we investigated whether the novel PI3K inhibitor HS-173 could attenuate radiation-induced up-regulation of DNA damage repair processes and assessed its efficacy as a radio- and chemo-sensitizer. Radiosensitizing effects of HS-173 were tested in human pancreatic cells using clonogenic survival and growth assays. Mechanisms underlying the effects of HS-173 and radiation were determined by assessing cell cycle and DNA damage- repair pathway components, including ataxia-telangiectasia mutated (ATM) and DNA-dependent protein kinase catalytic subunit (DNA-PKcs). The *in vivo* efficacy of HS-173 in cancer radiotherapy was evaluated using a human tumor xenograft model. HS-173 significantly increased the sensitivity of pancreatic cancer cells to radiation, an effect that was associated with G2/M cell cycle arrest. HS-173 also significantly attenuated DNA damage repair by potently inhibiting ATM and DNA-PKcs, the two major kinases that respond to radiation-induced DNA double-strand breaks (DSBs), resulting in sustained DNA damage. Moreover, the combination of HS-173 and radiation delayed tumor growth and impaired DNA repair in a pancreatic cancer xenograft model, reflecting enhanced radiosensitization. These results showed that HS-173 significantly improved radiotherapy by inhibiting the DNA damage-repair pathway in pancreatic cancer. We therefore suggest that HS-173 may be an effective radiosensitizer for pancreatic cancer.

## INTRODUCTION

Pancreatic cancer, one of the most lethal human cancers, is currently the fourth-most common cause of cancer-related death, and is estimated to be the second deadliest cancer by 2030 [1]. Recent increases in our understanding of cancer biology and the development of new treatment modalities, such as targeted therapy, immunotherapy and advanced radiologic techniques have helped to improve cancer survival. However, pancreatic cancer is still characterized by very poor prognosis with a 1 year overall survival rate of only about 10% and a 5-year overall survival rate of less than 5% [2]. Furthermore, no significant improvements in survival have been achieved during the last three decades (5-year survival, 4.4%), mainly owing to a lack of appropriate treatment modalities and proper methods for detecting pancreatic cancer in the early stage [3]. Less than 20% of patients are candidates for surgical resection; therefore, chemotherapy and radiation therapy remain the only treatment options [4].

To date, it has been estimated that, among cancer patients who have been cured, about 40% were treated with radiotherapy, with or without other modalities [5]. In the case of pancreatic cancer, patients with nonmetastatic pancreatic cancer benefit from the combination of radiation therapy and chemotherapy but radiation therapy in advanced-stage pancreatic cancer has remained in a matter of debate. In a Phase II study of induction chemotherapy followed by concurrent chemoradiotherapy in locally advanced pancreatic cancer patients, Kim *et al.* reported only modest survival gain [6]. This weak effect of radiation therapy in pancreatic cancer was mainly attributable to resistance to radiotherapy. The exact mechanism of radiation resistance has not yet been elucidated, but activity of the DNA damage-repair system is a problem that commonly underlies interruption of cancer radiotherapy. Accordingly, many researchers have investigated strategies for inhibiting DNA damage-response pathways. If radiation resistance could be overcome using radiosensitizers, it would enhance the efficacy of radiotherapy and increase survival, allowing initially unresectable primary tumors to be removed after radiotherapy in locally advanced pancreatic cancer. Therefore, new therapeutic options for overcoming radioresistance and enhancing radiotherapy are needed.

The phosphatidylinositol-4,5-bisphosphate 3-kinase (PI3K) pathway plays a key role in cell growth, proliferation and survival and is known to be disrupted in many cancers, including pancreatic cancer. Radiation also activates the PI3K/AKT pathway, an effect that is associated with radiotherapy resistance. Inhibition of the PI3K/AKT pathway has been shown to increase radiosensitivity in various cancers including glioblastoma, non-small cell lung cancer, colorectal cancer and head and neck squamous cell carcinoma [7–10]. A number of studies have shown that PI3K inhibitors exert synergistic effects in combination with radiation, resulting in improved radiosensitization [11–13]. Additionally, PI3K/AKT targeting led to an inhibition of repair response of radiation-induced DNA-double strand breaks (DSBs) and subsequent enhancement of radiation sensitivity [14]. In particular, this enhanced radiosensitivity is at least partly attributable to catalytic inhibition of ATM and DNA-PKcs enzymes, the main mediators of the DNA damage-repair system [15]. Since inhibition of the PI3K/AKT pathway increases therapeutic efficacy and helps to overcome radioresistance, targeting the PI3K/AKT pathway is an effective strategy for improving radiotherapy in cancer treatment. We previously reported that HS-173, novel PI3K inhibitor, has shown therapeutic effect against pancreatic cancer cells and xenograft models [16–18]. Here, we determined whether HS-173 enhances radiation sensitivity and anti-cancer effect by inhibiting DNA damage-repair processes in human pancreatic cancer. We demonstrated that inhibition of the PI3K/AKT pathway by HS-173 blocked DNA damage-repair signaling through suppression of ATM and DNA-PKcs activation, leading to radiosensitization *in vitro* and *in vivo*.

## RESULTS

### The radiosensitizing effects of HS-173 on pancreatic cancer cells

**Figure 1:**
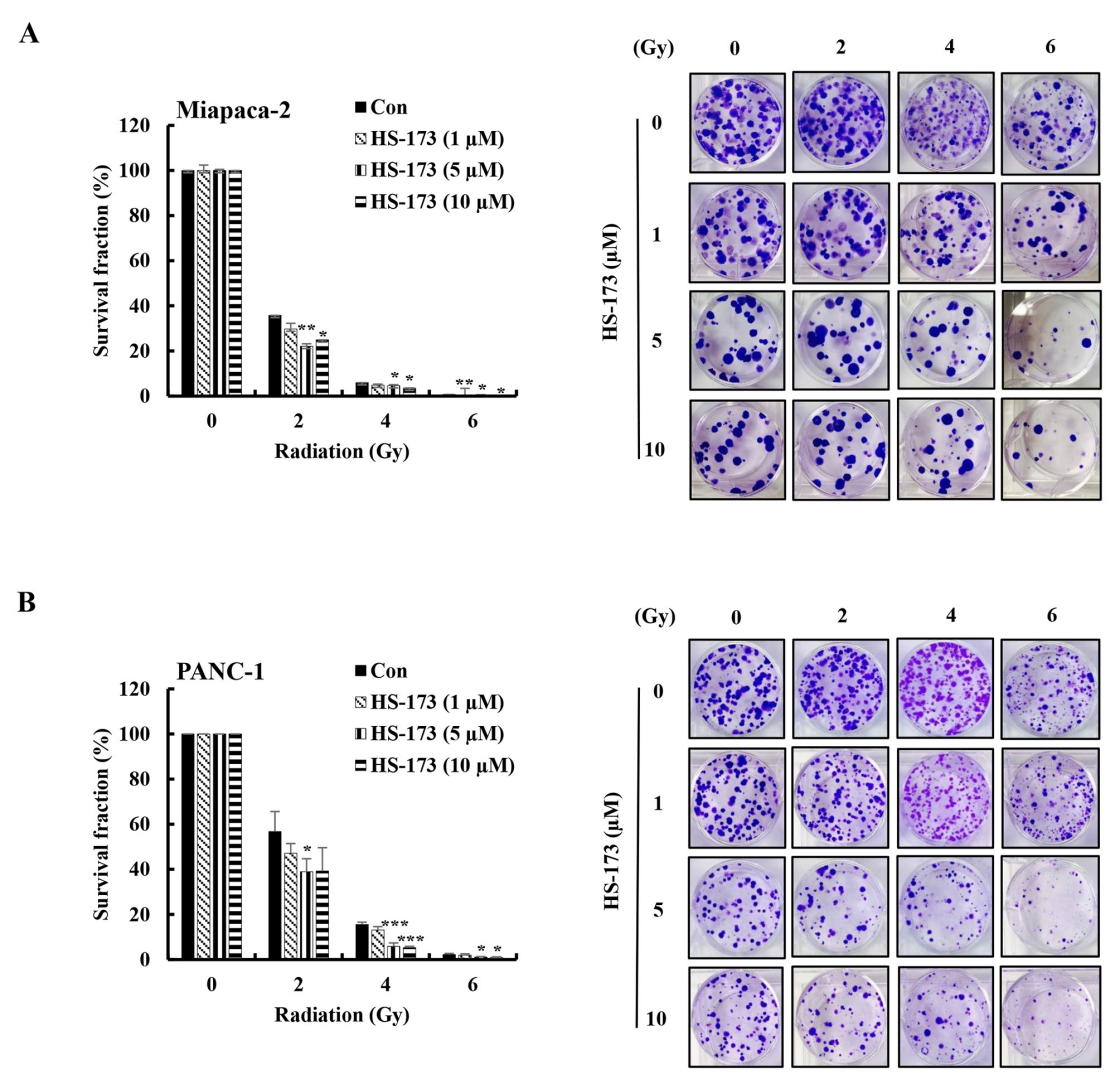
HS-173 significantly radiosensitizes pancreatic cancer cells Miapaca-2 **(A)** and PANC-1 **(B)** pancreatic cancer cells were treated with different concentrations of HS-173 for 24 h and then irradiated with the indicated doses (0–6 Gy). After 2 h, media were changed and cells were processed for clonogenic survival assays at the end of experiments (10-14 days). Colonies were counted by eye, using a cut-off value of 50 viable cells per colony. Data are presented as means ± S.D. from triplicate experiments (^*^P < 0.05, ^**^P < 0.01, and ^***^P < 0.001 vs. control).

To determine whether HS-173 might enhance radiosensitivity, we treated Miapaca-2 and PANC-1cells with HS-173, exposed them to different doses of radiation (2-6 Gy) for 24 h and then performed clonogenic assays. As shown in Figure 1, radiation caused a dose-dependent reduction in clonogenic survival in pancreatic cancer cells. Treatment with HS-173 for 24 h prior to irradiation resulted in a significant reduction in the clonogenic survival of pancreatic cancer cells at radiation doses of 2, 4, and 6 Gy, indicating that HS-173 induced synergic effects by enhancing the efficacy of radiation.

### Induction of apoptotic cell death by the combination of HS-173 and radiation

**Figure 2:**
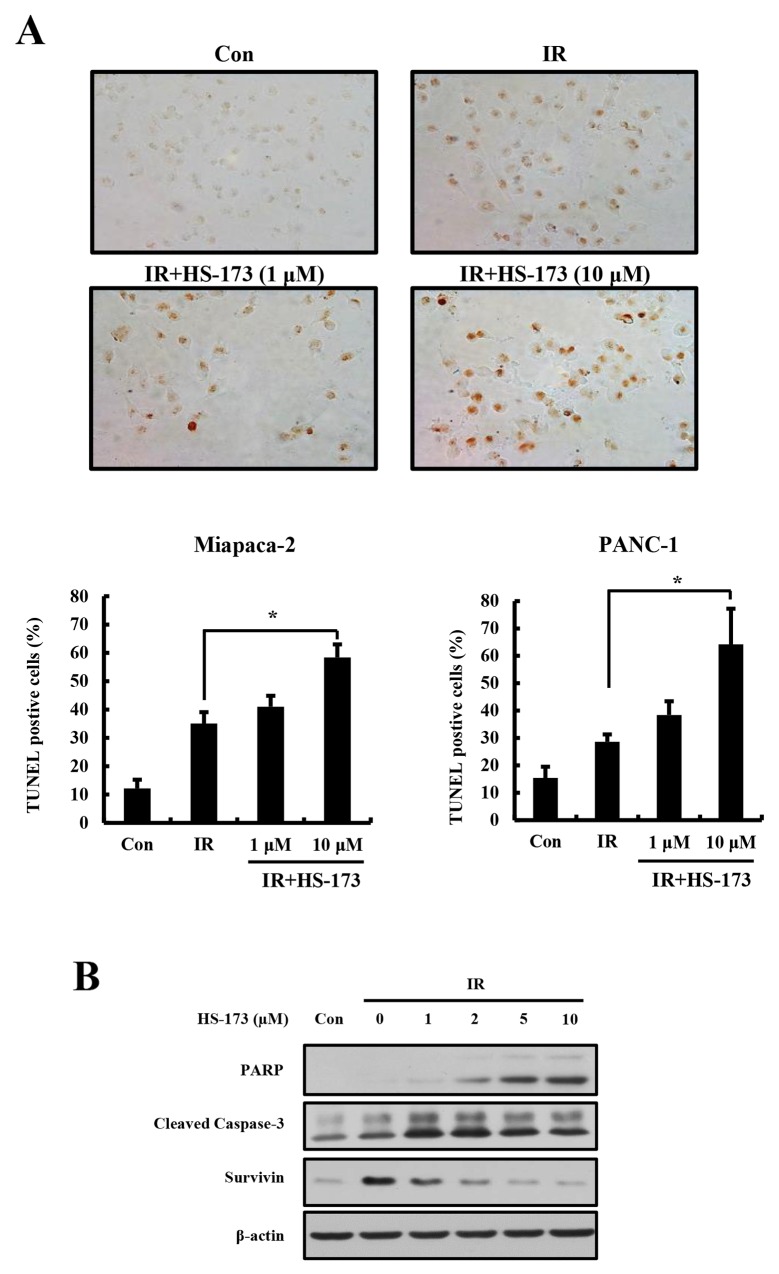
The combination of HS-173 and radiation synergistically induces apoptosis **(A)** Miapaca-2 and PANC-1 cells were pretreated with HS-173 (0-10 μM) for 6 h and then irradiated (4 Gy). After 24 h, cells were fixed and analyzed for apoptotic cell death by TUNEL assay. **(B)** Miapaca-2 cells were treated with HS-173 for 6 h and then irradiated (10 Gy). After 24 h, cell lysates were prepared and analyzed by Western blotting for cleaved PARP, cleaved caspase-3, and survivin. Data are expressed as means ± S.D. from three experiments (^*^P < 0.05).

Our previous studies showed that HS-173 induces apoptotic cell death in various cancer cell types [16–18]. Because combined treatment with HS-173 and radiation significantly reduced cell survival, we next investigated the apoptotic effects of this combination in pancreatic cancer cells using TUNEL (terminal deoxynucleotidyl transferase dUTP nick-end labeling) assays. For these experiments, we pretreated Miapaca-2 and PANC-1 cells with HS-173 (1 and 10 μM) for 6 h before irradiation, and then exposed the cells to radiation (10 Gy). In accord with clonogenic assays, radiation induced apoptotic cell death, as evidence by an increase in TUNEL-positive cells (Figure 2). Notably, combined treatment with HS-173 and radiation resulted in more apoptotic cell death compared with radiation alone. These results were confirmed by an assessment of apoptosis-related proteins, which showed that combined treatment with HS-173 and radiation increased the levels of cleaved caspase-3 and cleaved PARP, but decreased the expression levels of the anti-apoptotic survivin compared with radiation alone, demonstrating that the HS-173-induced increase in radiosensitivity resulted in an increase in apoptosis in pancreatic cancer cells.

### G2/M abrogation by the combination of HS-173 and radiation

**Figure 3:**
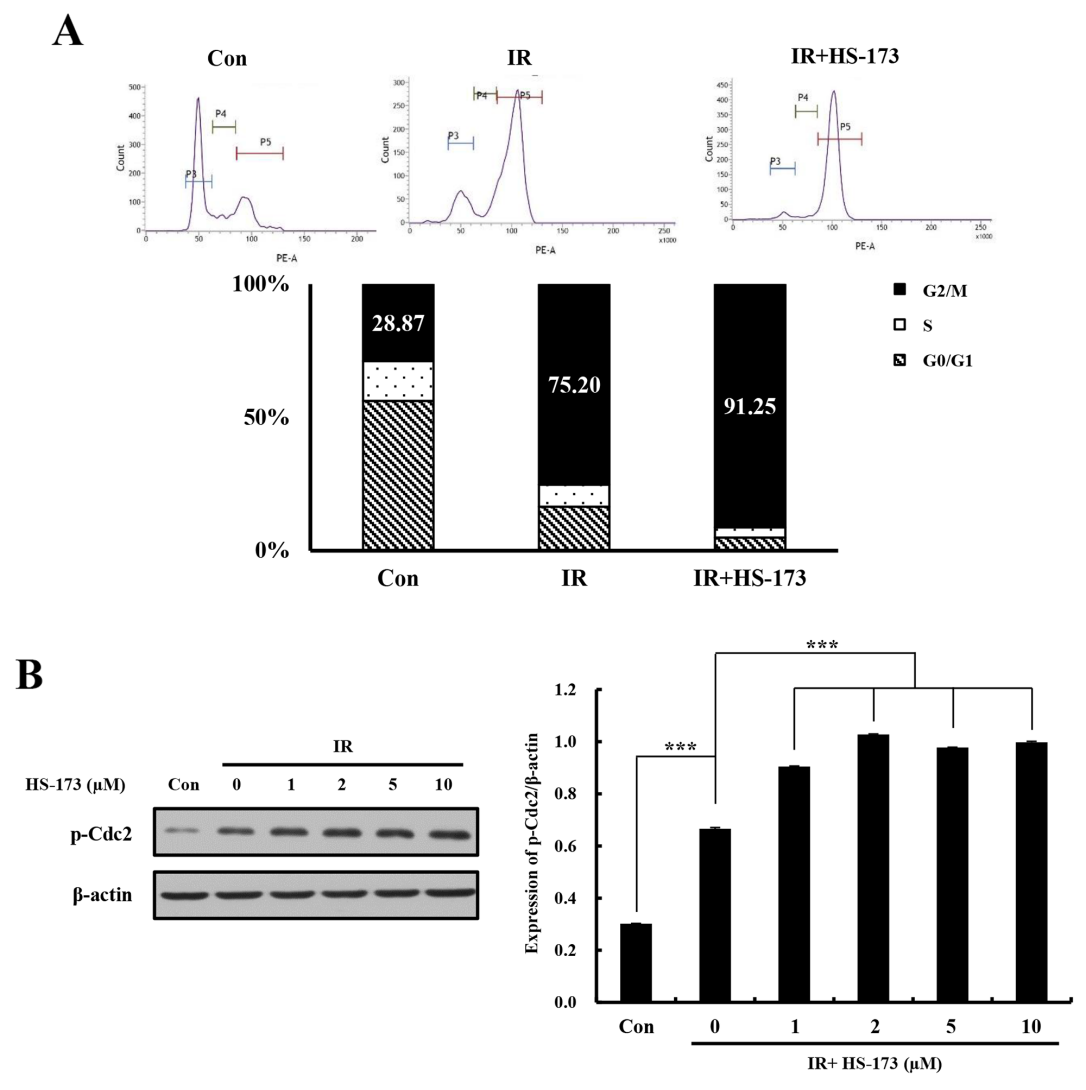
The combination of HS-173 and radiation increases G2/M arrest **(A)** Miapaca-2 cells were treated with HS-173 (0-10 μM) for 6 h before irradiation (10 Gy). After 24 h, cells were stained with PI and analyzed by flow cytometry. Quantitative PI staining data is presented as a percentage of the cell cycle distribution. **(B)** Miapaca-2 cells were treated with HS-173 for 6 h before irradiation (10 Gy). After 24 h, p-Cdc2 levels were assayed by Western blotting. Data are expressed as means ± S.D. from three experiments (^***^P < 0.001).

To investigate the mechanisms by which the combination of HS-173 and radiation caused radiosensitization in pancreatic cancer cells, we analyzed the cell cycle distribution after combined treatment. On the basis of our previous study [16], we hypothesized that HS-173 would cause radiation-treated cells to accumulate in G2/M phase. As expected, treatment of Miapaca-2 cells with radiation for 24 h increased the G2/M phase cell population; when combined with HS-173, radiation induced a greater degree of G2/M arrest (91%) than radiation alone (75%) (Figure 3A). A further investigation showed that expression of p-Cdc2, which typically causes cell cycle arrest in the G2/M phase of cell cycle, was increased to a greater extent in these cells by combined treatment than by radiation alone (Figure 3B). Collectively, these results indicate that the combination of HS-173 with radiation exerted synergistic cell cycle effects, causing accumulation of cells in G2/M phase, with an attendant delay in cell division.

### Increased expression of γ-H2AX and inhibition of ATM by HS-173 in irradiated pancreatic cancer cells

**Figure 4:**
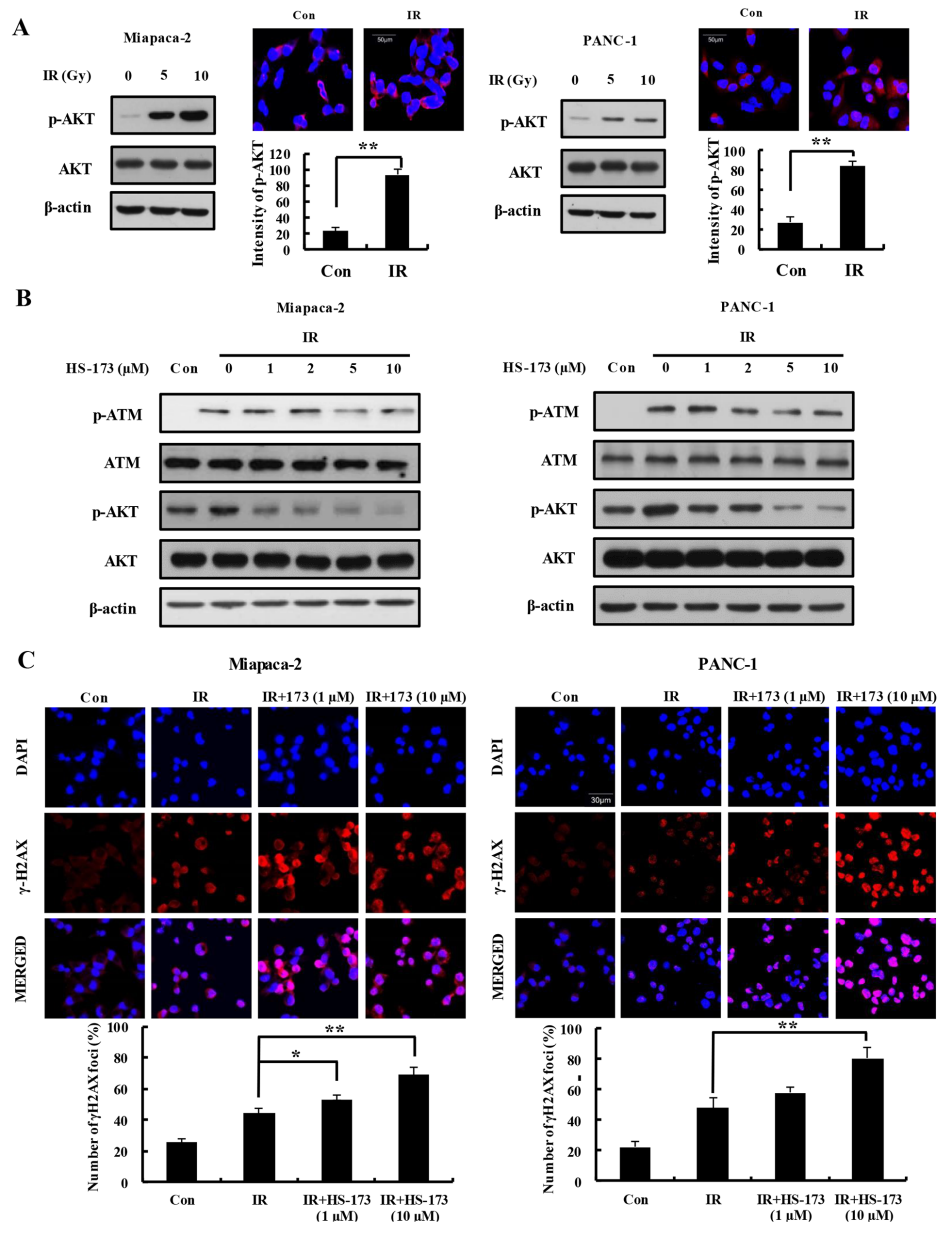
HS-173 increases expression of γ-H2AX through inhibition of ATM in irradiated pancreatic cancer cells **(A)** Pancreatic cancer cells were irradiated with the indicated dose (5 and 10 Gy). In radiation condition, the high expression of p-AKT was observed in pancreatic cancer cells using Western blotting and immunoflorescence. **(B)** Miapaca-2 and PANC-1 cells were exposed to radiation (10 Gy) alone and/or together with different concentrations of HS-173 for 6 h. p-ATM and p-AKT levels were assessed by Western blotting. **(C)** Miapaca-2 and PANC-1 cells were pretreated with HS-173 for 6 h, irradiated (4 Gy) for 30 min, and then immunostained for γ-H2AX. γ-H2AX staining was quantified by analyzing the optical density of stained cells. Data are expressed as means ± S.D. from three experiments (^*^P < 0.05 and ^**^P < 0.01).

Radiation has been shown to induce PI3K/AKT activation in many cell types, and activation of the PI3K/AKT signaling pathway is correlated with radioresistance [19–20]. Accordingly, we assessed activation of AKT by comparing the levels of phosphorylated AKT (p-AKT) in Miapaca-2 and PANC-1 pancreatic cancer cells after treatment with 5 and 10 Gy radiation. Immunofluorescence analyses using antibodies specific for p-AKT confirmed that radiation increased expression of p-AKT in both Miapaca-2 and PANC-1 cells, implicating AKT in the cellular response to radiation in pancreatic cancer cells (Figure 4A). ATM is a principal regulator of the DNA damage response following irradiation [21]. In particular, ATM is a major determinant of AKT activation in response to radiation [22]. Thus, we investigated whether HS-173 was capable of inhibiting radiation-induced activation of ATM and AKT. Miapaca-2 and PANC-1 cells were pretreated with HS-173 for 6 h and then the cells were incubated for 30 min after radiation treatment (10 Gy). As shown in Figure 4B, HS-173 significantly attenuated radiation-induced increases in the levels of p-AKT and p-ATM, indicating that HS-173 inhibited critical components of the DNA damage response in human Miapaca-2 and PANC-1 pancreatic cancer cells. In addition, HS-173 increased the expression of γ-H2AX, which is frequently used as a marker for DNA double-strand breaks (DSBs) following irradiation (Figure 4C).

### Impairment of the DSB repair response by HS-173 through inhibition of DNA-PKcs in irradiated pancreatic cancer cells

**Figure 5:**
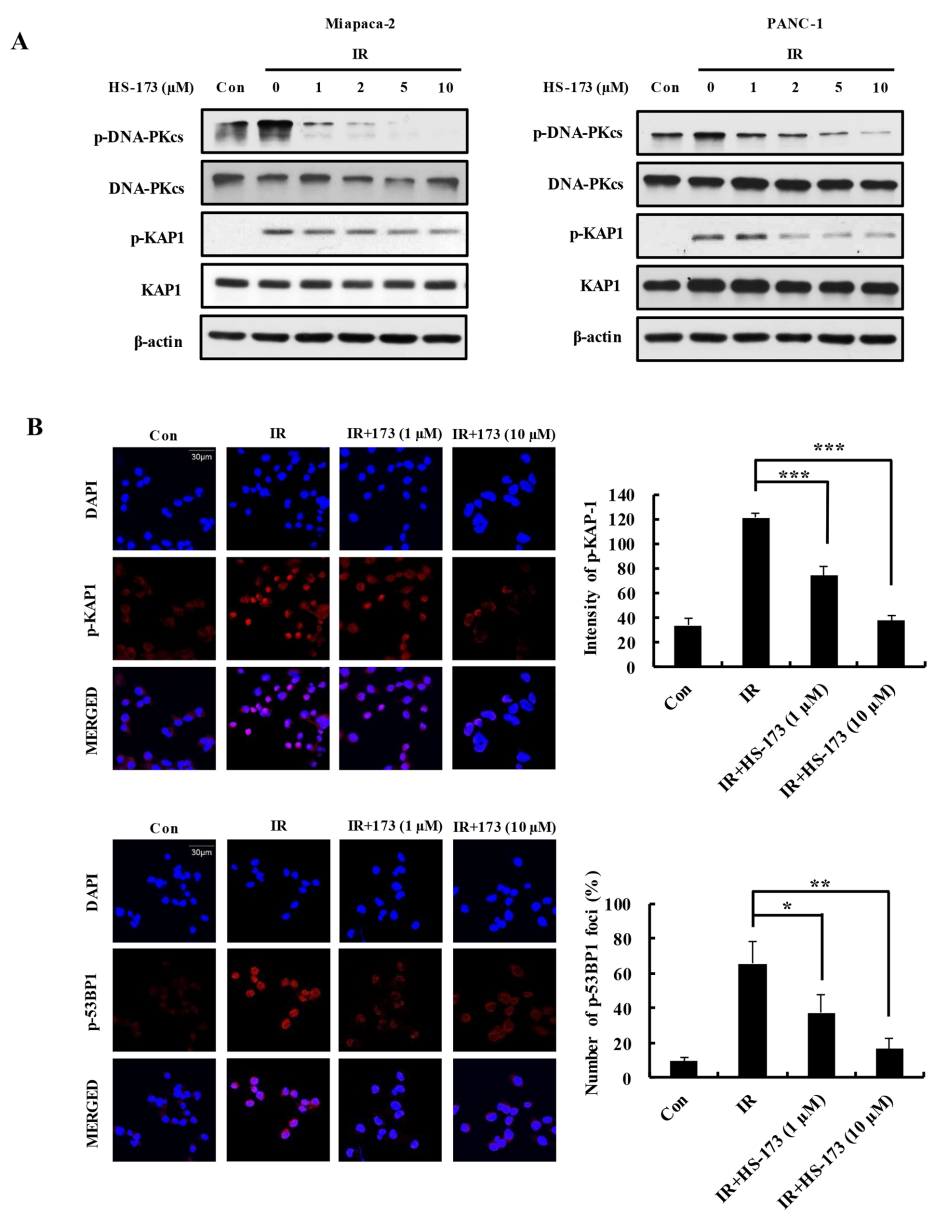
HS-173 impairs DSB repair responses by inhibiting DNA-PKcs activation in irradiated pancreatic cancer cells **(A)** Miapaca-2 and PANC-1 cells were pretreated for 6 h with or without different concentrations of HS-173, and then exposed to radiation (10 Gy) for 30 min. p-DNA-PKcs, p-KAP1, and p-53BP1 levels were determined by Western blotting. **(B)** Miapaca-2 and PANC-1 cells were pretreated for 6 h with or without different concentrations of HS-173, then exposed to radiation (4 Gy) for 30 min. Cells were then stained for p-KAP1 and p-53BP1 (red) by immunofluorescence, and immunostaining was quantified densitometrically. Data are expressed as means ± S.D. from three experiments (^*^P < 0.05, ^**^P < 0.01 and ^***^P < 0.001).

Because PI3K/AKT signaling promotes DSB repair in various cancers primarily through processes involving DNA-PKcs [22–25], we assessed whether HS-173 radiosensitized human pancreatic cancer cells by inhibiting DNA repair. To this end, we treated Miapaca-2 and PANC-1 pancreatic cells with different concentrations of HS-173 for 6 h, harvested cells after 30 min exposure to radiation, and determined levels of DNA-PKcs, KAP1, and 53BP1 by Western blotting analysis. We found that HS-173 inhibited radiation-induced activation of DNA-PKcs in pancreatic cancer cells in dose-dependent manner, thereby blocking phosphorylation of the downstream substrates, KAP1, and 53BP1, a marker for DSBs (Figure 5A). Inhibition of KAP1 and 53BP1 phosphorylation was confirmed by immunostaining (Figure 5B). Collectively, these findings indicate that HS-173 inhibits DNA-PKcs and severely impairs the DSB repair response in irradiated pancreatic cancer cells.

### Delayed tumor growth by radiosensitization and inhibition of DNA repair responses through HS-173 in irradiated pancreatic cancer xenograft models

**Figure 6:**
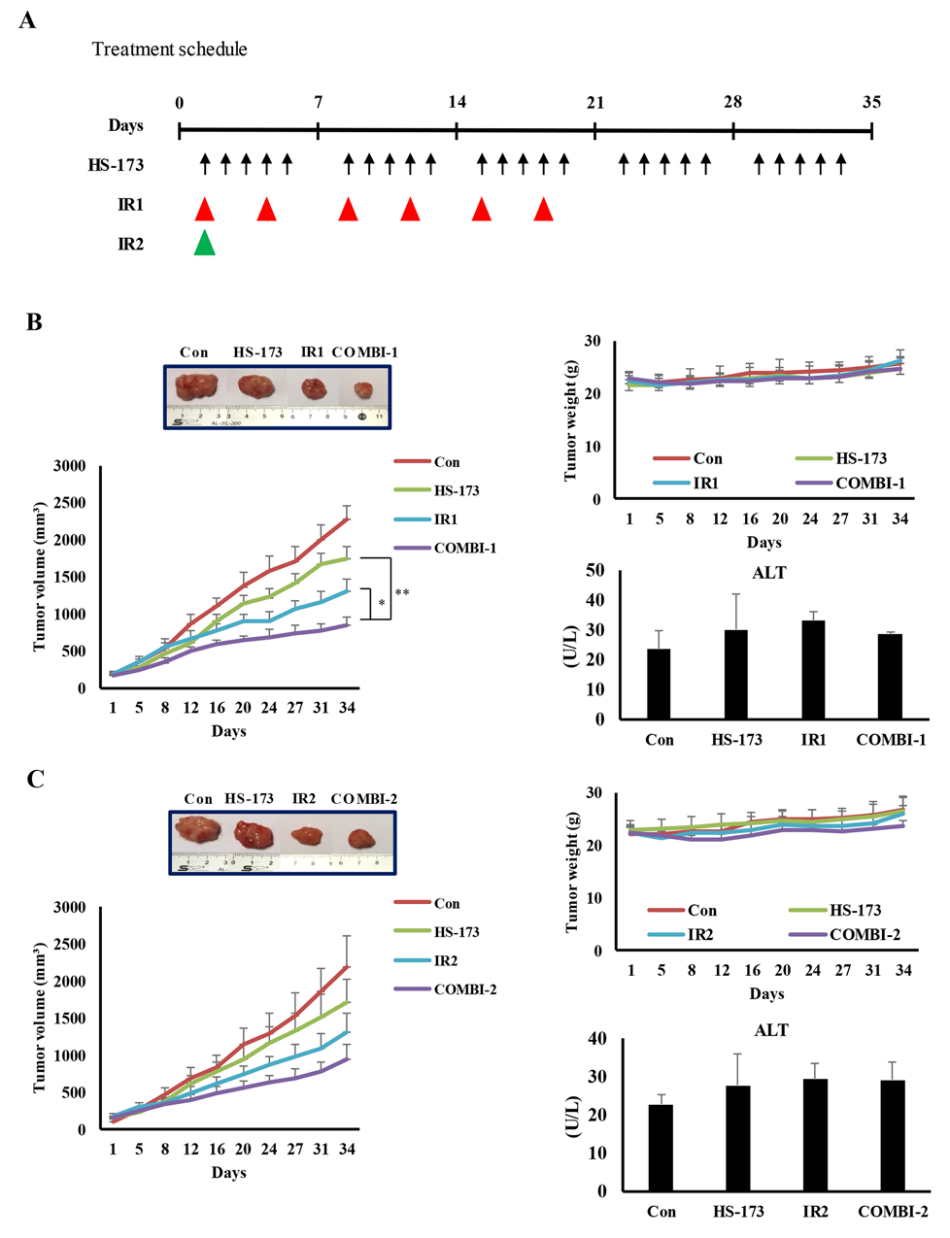

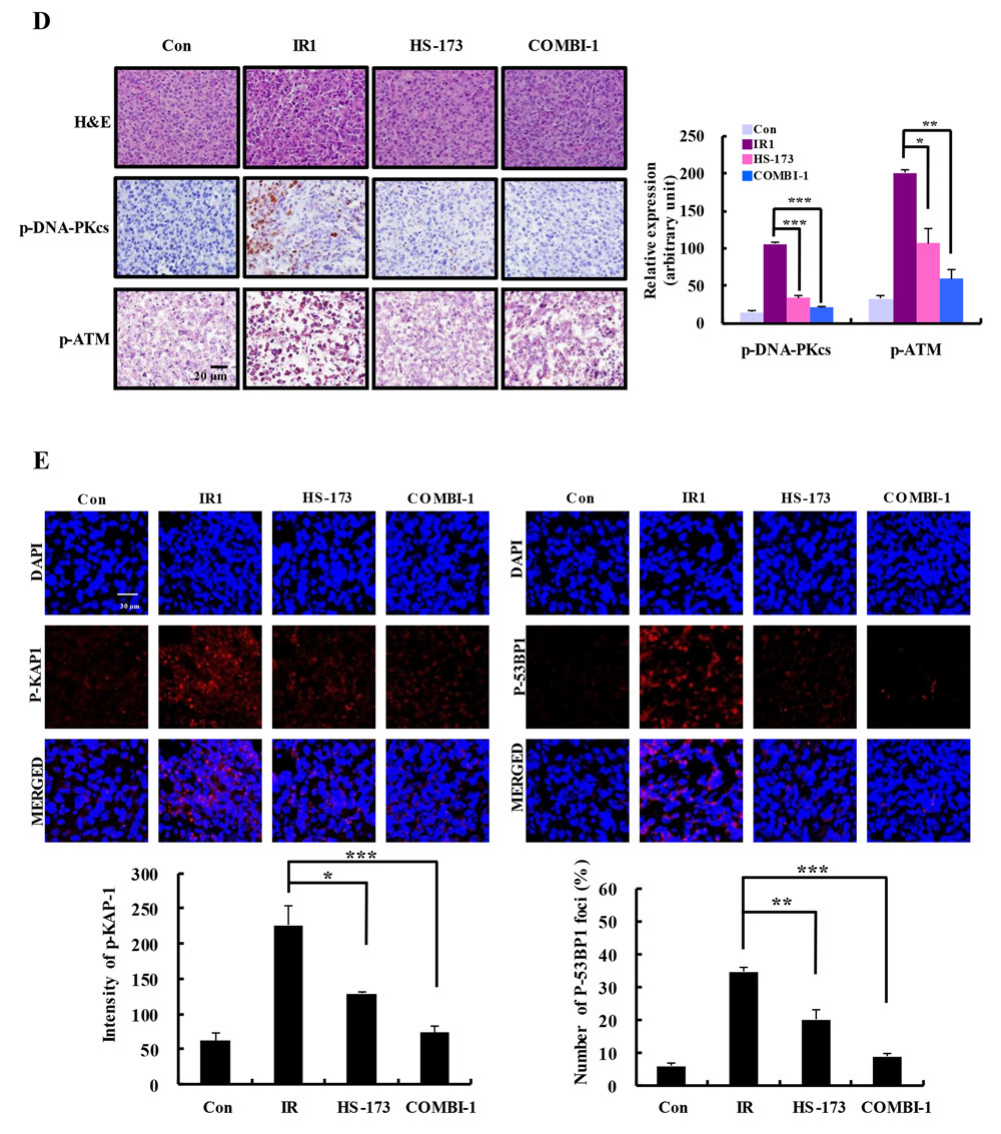
HS-173 inhibits tumor growth and DNA repair responses, thereby inducing radiosensitization in irradiated xenograft models **(A)** Experiment schedule. Nude mice were intraperitoneally administered HS-173 (10 mg/kg, 5 times per week) and then irradiated (IR1: 2 Gy, twice a week for 3 weeks; IR2: 8 Gy, once). **(B and C)** Tumor size and body weight were measured every 2 days. After 34 days, tumors were excised and blood was collected for evaluation of ALT levels. A set of tumors excised at the end of the treatment period illustrates the marked reduction in tumor size with combination treatment. **(D and E)** Isolated pancreatic tumors were sectioned (8-μm) and stained for p-ATM, p-53BP1 and p-KAP1 by immunofluorescence and immunohistochemistry. Data are presented as means ± S.D. (^*^P < 0.05, ^**^P < 0.01, and ^***^P < 0.001).

To determine if inhibition of DSB repair by HS-173 results in radiosensitization and tumor regression, we generated subcutaneous tumors using Miapaca-2 pancreatic cancer cells, and allowed the tumors to grow a to volume of 100 mm^3^ before initiating treatment. To compare the ability of either a single dose or a fractionated dose schedule of local ionizing radiation to control the growth of tumors, we exposed mice, with or without HS-173 (10 mg/kg) pretreatment, to two radiation therapy schedules: IR1, six exposures to 2 Gy; and IR2, a single exposure to 8 Gy (Figure 6A). Using the IR1 paradigm (fractionated dose), tumor growth was modestly delayed in mice treated with radiation alone compared with the control group; HS-173 alone also inhibited tumor growth. In contrast, combined treatment with ionizing radiation and HS-173 dramatically enhanced radiosensitization, as evidenced by a significantly greater inhibition of tumor growth. Mice treated with both HS-173 and radiation also showed no obvious changes in body weight or ALT (alanine transaminase) levels, indicating that this combination treatment is relatively nontoxic (Figure 6B). Combination treatment also caused enhanced inhibition of tumor growth in the IR2 paradigm (single dose) (Figure 6C). To determine if the tumor regression caused by combination treatment was associated with a DSB repair response, we assessed the expression of the DSB repair pathway-related proteins, ATM, DNA-PKcs and KAP1. As shown in Figure 6D, the combination of HS-173 and radiation significantly inhibited the phosphorylation of ATM and DNA-PKcs. Consequently, the combination treatment showed a significant reduction in phosphorylation of KAP1 and 53BP1, a marker for DSB repair (Figure 6E), implying that inhibition of ATM and DNA-PKcs is sufficient to inhibit downstream signaling through KAP-1 in pancreatic tumors.

## DISCUSSION

Despite decades of research, pancreatic cancer remains among the most lethal tumors for which no effective treatment exists [26]. Because of the atypical clinical symptoms of pancreatic cancer, most patients are diagnosed too late to have the opportunity of surgery to remove the cancer [27]. Radiotherapy and/or chemotherapy are the common methods used to manage patients with local advanced pancreatic cancer and prolong survival. Radiotherapy is the treatment of choice for pancreatic cancer, but the effectiveness of treatment is limited by radiation resistance. Given that pancreatic cancer show high rate of resistance to radiotherapy, it needs novel therapeutic approaches to enhance radiotherapy for pancreatic cancer treatment. The PI3K/AKT pathway is uniformly activated in human pancreatic ductal adenocarcinoma and mouse models of K-Ras-driven pancreatic cancer [28]. Additionally, this pathway is highly associated with radiotherapy resistance [29]. Given the identification of the PI3K/AKT axis as a potential radiation-modulation target in cancer, we performed a preclinical investigation of the radiosensitizing effects of the PI3K inhibitor HS-173. We found that inhibition of PI3K/AKT by HS-173 treatment significantly radiosensitized pancreatic cancer to the subsequent effects of radiation, as demonstrated by the increase in apoptosis and altered cell cycle resulting from inhibition of the DNA repair response.

The PI3K pathway is commonly activated in various human cancers including pancreatic cancer [30]. In particular, K-Ras mutation has been found in more than 90% of pancreatic cancer patients [31], which activate Ras-dependent downstream effectors pathways including PI3K signaling. Many studies have reported that inhibition of the PI3K pathway causes cell cycle arrest, apoptosis, and decreased cell growth [32–34]. Therefore, we tested the hypothesis that inhibition of the PI3K/AKT pathway by HS-173 would sensitize pancreatic cancer cells to radiation. Indeed, we showed that the combination of HS-173 and radiation synergistically increased therapeutic efficacy by blocking cell proliferation compared with either treatment alone (Figure 1). HS-173 also enhanced radiation-induced apoptosis, as evidenced by increased levels of cleaved PARP and caspase-3 as well TUNEL-positive cells. Some studies have shown that the combination of a PI3K inhibitor and radiation or chemotherapy can improve the efficacy of both therapies [11, 15, 35]. Georgia *et al.* reported that the PI3K inhibitor BEZ-235 exerted synergistic effects with chemoradiation in the treatment of non-small lung cancer harboring K-Ras mutations [15].

We further found that the combination of HS-173 and radiation significantly increased the proportion of cells in G2/M phase and reduced the proportion of cells in S phase. The synergic effects of HS-173 and radiation not only inhibited the proliferation of pancreatic tumor cells, they also caused arrest of living cells in the G2/M phase of the cell cycle. These effects were confirmed by the demonstration that combined treatment increased the expression of p-Cdc2 in Miapaca-2 cells, in associated with delayed cell division. The G2/M phase plays a very important role in the synthesis and repair of DNA. Cells arrested in this phase resist proliferation and differentiation, but are also unable to repair the DNA damage induced by chemotherapy and radiation [35, 36]. The synergistic effect of HS-173 and radiation on the induction of cell cycle arrest in G2/M phase could enhance apoptosis in pancreatic cancer cells. Thus, HS-173 may exert its biological effects by promoting apoptosis and blocking the cell cycle in the G2/M phase, preventing cells from entering the next cycle and thereby inhibiting cell proliferation.

Induction of G2/M arrest by radiation was initially considered a passive consequence of damaged DNA, but extensive subsequent investigations have led to speculation that DNA repair plays an active role in this process [37, 38]. Indeed, a long G2 delay in a DNA repair-proficient background has been linked to radioresistance [39]. In G2-irradiated cells, about 20% of IR-induced DSBs are repaired with slow kinetics representing those DSBs that undergo resection and repair [40]. Accordingly, even if radiation induces greater DNA damage, radioresistance is inevitable if DNA repair occurs more rapidly than DNA damage accumulation. Accordingly, several studies have validated the effects of radiosensitizers in inhibiting DNA damage repair signaling pathways—including those involving ATM and DNA-PKcs—both *in vitro* and *in vivo*. Attenuation of the PI3K/AKT pathway increases therapeutic efficacy in radioresistant cancers [41, 42]. This has been demonstrated using several different PI3K inhibitors that can inhibit the DNA damage repair process. Indeed, it has been shown that the PI3K/AKT inhibitor BEZ-235 inhibits DNA damage response proteins, including ATM and DNA-PKcs, in glioma [15], and the PI3K inhibitor LY294002 enhances the efficacy of radiotherapy in cervical cancer [43]. As noted above, a major component of this radiosensitization is secondary to off-target effects through DNA-PKcs and ATM [15, 44]. In this study, we found that HS-173 inhibited radiation-induced activation of both ATM and DNA-PKcs, the two major kinases that respond to DSBs. HS-173 also inhibited expression of P-KAP1, downstream target of these kinases, in irradiated pancreatic cancer cells. Cross-inhibition of ATM and DNA-PKcs by PI3K inhibitors is not surprising because these kinases have homologous catalytic domains [45, 46]. In accord with our results, a previous study reported that the PI3K inhibitor BEZ-235 radiosensitized non-small cell lung carcinoma cells expressing oncogenic K-Ras, an effect that was correlated with higher levels of radiation-induced DNA breaks [47, 15]. ZSTK474, another PI3K inhibitor, has also been shown to inhibit DNA-PKcs activity [48]. We further observed that HS-173 inhibited tumor growth by enhancing the efficacy of radiation through inhibition of elements of the DNA damage repair response pathway, including ATM and DNA-PKcs. Gupta *et al.,* examining radiosensitization by the PI3K inhibitor LY294002 in bladder cancer xenograft models, reported similar results [49].

In this study, we demonstrated that HS-173 is a potent inhibitor of PI3K/AKT signaling that responds to radiation-induced DNA breaks and repair by inhibiting ATM and DNA-PKcs, resulting in profound radiosensitization in pancreatic cancer cells. Moreover, the radiosensitizing effects of HS-173 appear to be associated with G2/M cell cycle arrest and induction of apoptosis in irradiated cells. Therefore, we suggest that HS-173 could be a potential radiosensitizer option for pancreatic cancer in combination with radiotherapy.

## MATERIALS AND METHODS

### Cells and materials

The human pancreatic cancer cell lines, PANC-1 and Miapaca-2, were purchased from the American Type Culture Collection (ATCC, Manassas, VA, USA). PANC-1 and Miapaca-2 cells were cultured in Dulbecco's Modified Eagle Medium (DMEM) supplemented with 10% fetal bovine serum (FBS) and 1% penicillin/streptomycin. Cell culture media, FBS, penicillin-streptomycin, and other supplementary reagents were purchased from GIBCO (Uxbridge, UK). All cell lines were maintained at 37°C in a CO_2_ incubator with a controlled humidified atmosphere composed of 95% air and 5% CO_2_.

### Preparation of HS-173

The imidazopyridine derivative, ethyl 6-(5-(phenylsulfonamido)pyridin-3-yl)imidazo[1,2-a]pyridine-3-carboxylate (HS-173), is a new PI3Kα inhibitor that was synthesized as described in our previous study [16]. For all *in vitro* studies, HS-173 was dissolved in dimethylsulfoxide (DMSO) at a concentration of 10 mM before use.

### Irradiation

Cells were irradiated with γ-rays using a ^137^Cs irradiation source (Model 68; J.L. Shepherd and Associates, Glenwood, CA, USA) at a dose rate of 3-4 Gy/min.

### Clonogenic assay

Pancreatic cancer cells were plated at appropriate densities in collagen-coated 6-well plates and incubated overnight. Cells were treated with different concentrations of HS-173 for 24 h, and then irradiated with the indicated doses. After a 2 h exposure to radiation, the medium was changed and cells were cultured for 14 days in a 5% CO_2_ incubator at 37°C. Colonies were fixed with 4% paraformaldehyde (PFA) and stained with 0.5% crystal violet. Colonies containing more than 50 cells were counted, and averages of triplicate dishes were obtained for each sample. The results were normalized with respect to the plating efficiencies of the corresponding non-irradiated cells, and the surviving fractions were calculated.

### Western blotting

After the cells were treated with different concentrations of HS-173 and irradiated for various times, they were collected and washed with cold phosphate-buffered saline (PBS). The cells were then lysed with RIPA buffer containing protease and phosphatase inhibitor cocktails (GenDEPOT, Barker, TX, USA). Proteins in whole-cell lysates were resolved by sodium dodecyl sulfate protease and phosphatase inhibitor (SDS-PAGE), and transferred onto nitrocellulose membranes. The blots were first incubated with the appropriate primary antibodies, and then with horseradish peroxidase (HRP)-conjugated secondary antibodies. Antibody binding was detected using enhanced chemiluminescence reagents (Bio-Rad, Hercules, CA, USA). Primary monoclonal antibodies against the following proteins were used: cleaved PARP, cleaved caspase-3, survivin, p-AKT, AKT (Cell Signaling Technology, MA, USA) p-Cdc2, p-ATM, ATM, p-DNA-PKcs, DNA-PKcs, p-KAP1, p-53BP1 (Abcam, Cambridge, UK) KAP1, 53BP1(Santa Cruz, Dallas, TX, USA) and β-actin (Sigma-Aldrich, OH, USA) Secondary antibodies were purchased from Santa Cruz Biotechnology (Dallas, TX, USA).

### TUNEL assay

Miapaca-2 and PANC-1 cells were pretreated with HS-173 (0-10 μM) for 6 h and then irradiated (4 Gy) for 24 h. After treatment, cells were fixed in ice-cold 4% PFA, washed with PBS, and then analyzed for apoptotic cells using a TUNEL assay kit (Chemicon, Temecula, CA, USA), following the manufacturer's instructions.

### Immunofluorescence

After treating with HS-173 for 6 h, Miapaca-2 and PANC-1 cells were irradiated with 4 Gy for 30 min, washed twice with PBS, and fixed by incubating in an acetone:methanol solution (1:2) for 10 min at −20°C. Fixed cells were incubated in blocking buffer for 1 h at room temperature, and then incubated overnight with primary antibody (p-AKT, γ-H2AX, p-KAP1, p-53BP1) at 4°C. After washing several times with PBS, cells were incubated with rhodamine isothiocyanate (RITC)-conjugated rabbit secondary antibody (1:100; Dianova, Hamburg, Germany) for 1 h at room temperature, and then counterstained with 4,6-diamidino-2-phenylindole (DAPI) to visualize nuclei. Slides were then washed twice with PBS, covered with the anti-fading agent, 1,4-diazobicyclo (2,2,2)-octane (DABCO, Sigma-Aldrich), and viewed with a confocal laser-scanning microscope (Olympus) at 488 and 568 nm.

### Flow cytometry

Miapaca-2 cells were treated with HS-173 (0-10 μM) for 6 h before irradiation (10 Gy), then fixed by incubating at −25°C overnight in cold 70% ethanol. After washing with PBS, the cells were stained with 50 μg/mL propidium iodide (PI) and 100 μg/mL RNase A for 30 min at room temperature in the dark, and then analyzed by flow cytometry to determine the percentage of cells in specific phases of the cell cycle using a FACS Calibur flow cytometer (BD Biosciences, San Jose, CA, USA). Flow cytometry data were analyzed using FlowJo software (Tree Star, Ashland, OR, USA).

### Immunohistochemistry

Tumor tissue was excised, cut into 8-μm-thick sections, deparaffinized, and subjected to antigen retrieval by microwaving in citrate buffer (pH 6.0) for 5 min. After quenching peroxidase with 0.3% hydrogen peroxide (H_2_O_2_) in PBS for 10 min, sections were washed in water and pre-blocked with normal goat or horse serum for 1 h. Tissue sections were then incubated overnight at 4°C with anti-p-ATM antibody (Abcam, Cambridge, UK), diluted 1:50. After washing with PBS, sections were incubated with biotinylated secondary antibodies (1:100) for 1 h, and then streptavidin-HRP was applied. Finally, immunoreactive proteins were developed by incubating sections with diaminobenzidine tetrahydrochloride substrate (DAB) for 10 min, followed by counterstaining sections with hematoxylin. At least three random fields of each section were examined at a magnification of ×400.

### Tumor xenograft studies

A mouse Miapaca-2 cells tumor xenograft model was established by injecting the right flank of 6-week old athymic BALB/c nude mice (Central Lab. Animal Inc. Seoul, Korea) with 5 × 10^6^ Miapaca-2 cells (in PBS). After tumors reached a volume of 50-100 mm^3^, mice were intraperitoneally administered HS-173 (10 mg/kg, 5 times/week) and then irradiated using fractionated dosing (IR1: 2 Gy, twice a week for 3 weeks) or a single dose (IR2: 8 Gy, once). Body weight and tumor dimensions were recorded twice per week. Tumor size was calculated according to the formula, 0.5 × long axis × (short axis)^2^. Animal care and experimental procedures were conducted in accordance with the Guide for Animal Experiments, published by the Korean Academy of Medical Sciences, and the protocols used were approved by the Institutional Animal Care and Use Committee of Inha University Hospital (Approval No. 151119-388-1).

### Statistical analysis

Data are expressed as means ± S.D., and were analyzed by analysis of variance (ANOVA) or unpaired Student's t-test, as appropriate. Duncan post hoc tests were applied to assess significant differences between groups. A p-value ≤ 0.05 was considered statistically significant. Comparisons of results were performed using Student's t-test.
